# A single factor dominates the behavior of rhythmic genes in mouse organs

**DOI:** 10.1186/s12864-019-6255-3

**Published:** 2019-11-20

**Authors:** Yang Cheng, Yuhao Chi, Luoying Zhang, Guang-Zhong Wang

**Affiliations:** 1 CAS Key Laboratory of Computational Biology, CAS-MPG Partner Institute for Computational Biology, Shanghai Institute of Nutrition and Health, Shanghai Institutes for Biological Sciences, University of Chinese Academy of Sciences, Chinese Academy of Sciences, Shanghai, China, Shanghai, 200031 China; 2 Key Laboratory of Molecular Biophysics of Ministry of Education, College of Life Science and Technology, Huazhong University of Science and Technology, Wuhan, 430074 Hubei China

**Keywords:** Circadian rhythm, Rhythmically expressed gene, Rhythmic gene function, Energy cost

## Abstract

**Background:**

Circadian rhythm, regulated by both internal and external environment of the body, is a multi-scale biological oscillator of great complexity. On the molecular level, thousands of genes exhibit rhythmic transcription, which is both organ- and species-specific, but it remains a mystery whether some common factors could potentially explain their rhythmicity in different organs. In this study we address this question by analyzing the transcriptome data in 12 mouse organs to determine such major impacting factors.

**Results:**

We found a strong positive correlation between the transcriptional level and rhythmic amplitude of circadian rhythmic genes in mouse organs. Further, transcriptional level could explain over 70% of the variation in amplitude. In addition, the functionality and tissue specificity were not strong predictors of amplitude, and the expression level of rhythmic genes was linked to the energy consumption associated with transcription.

**Conclusion:**

Expression level is a single major factor impacts the behavior of rhythmic genes in mouse organs. This single determinant implicates the importance of rhythmic expression itself on the design of the transcriptional system. So, rhythmic regulation of highly expressed genes can effectively reduce the energetic cost of transcription, facilitating the long-term adaptive evolution of the entire genetic system.

## Background

Circadian rhythm refers to a 24-h self-sustained oscillation of physiological processes, which is evolutionarily conserved [1–5]. In animals, this oscillation coordinates various physiological activities, including behaviors, such as sleeping and feeding [6–8]. Surprisingly, this oscillation exists not only in an individual organism as a whole, but is also widely detected in the constituting tissues and single cells [9–13]. Thousands of genes display rhythmic transcriptional oscillation, as has been determined by either microarray or RNA sequencing technologies [10, 12, 14–16]. On single-cell level, almost every cell utilizes these oscillations to control or regulate own overall gene expression [9, 17, 18], indicating that circadian regulation plays a fundamental role in the transcriptional system.

The core circadian transcriptional network in mammals consists of several important transcription factors, such as *Clock*, *Bmal1*, *Per1*/*Per2*, and *Cry1*/*Cry2*. Although the negative feedback loop in the circadian regulatory network is conserved in different tissues, the regulated genes in each tissue are distinct from each other. According to an early study involving microarray expression profiling, approximately 8–10% expressed genes are rhythmically regulated in the mouse liver and heart [10]. Importantly, rhythmically expressed genes in the two tissues rarely overlap, indicating that these genes are highly tissue-specific. Tissue specificity of rhythmic genes was subsequently widely confirmed. Zhang et al. [14] constructed a circadian gene expression atlas by using data for 12 mouse organs, and found that approximately half of the protein-coding genes are expressed rhythmically, with strong organ-specific signals. Similar, in humans, more than 7000 genes show rhythmic expression pattern in at least one of 13 tissues collected; 12% of these genes are drug targets [16]. A systematic study of 64 tissues from the baboon indicated that over 80% of protein-coding genes are rhythmically expressed across the body, with few overlapping [19]. The wide distribution of rhythmically regulated genes indicates their importance to the functional specificity of each tissue.

In addition to tissue specificity, rhythmically expressed genes also exhibit species-specific characteristics. A detailed comparison of 11 tissues in mouse and baboon suggested that only a small proportion of rhythmically expressed genes overlap in each tissue, and no significant correlation was observed between the numbers of rhythmic genes in the two species [19]. Further, only 46 out of 188 rhythmically expressed genes in the epidermis in humans exhibit strong oscillation (i.e., high amplitude and cycling) in the epidermis of mouse [15]. The organ- and species-specific characteristics of rhythmically expressed genes are two fundamental properties of the circadian regulatory network. These properties indicate that the factors that affect the distribution pattern of rhythmic genes may be very complex. However, it is unclear whether a single dominant factor exists.

Here, we aimed to determine whether a single major factor exists, that influences the expression of rhythmic genes. By investigating all rhythmically expressed genes in the circadian gene expression atlas [14], which to date is the largest circadian atlas for mouse, we have identified expression level as the key factor that dominates rhythmic gene expression. It explained the majority of variations in the circadian amplitude of cyclic genes in 12 mouse organs. We also examined the role of gene function and tissue specificity in rhythmic expression. Finally, we surveyed the energy consumed during the expression of different regions of cyclic genes and explored its effects on rhythmic expression. Overall, the presented data suggest that a unified model can potentially be used to explain rhythmic gene expression in various mouse organs.

## Results

### Gene expression level explains > 70% of the variation in amplitude

**Fig. 1:**
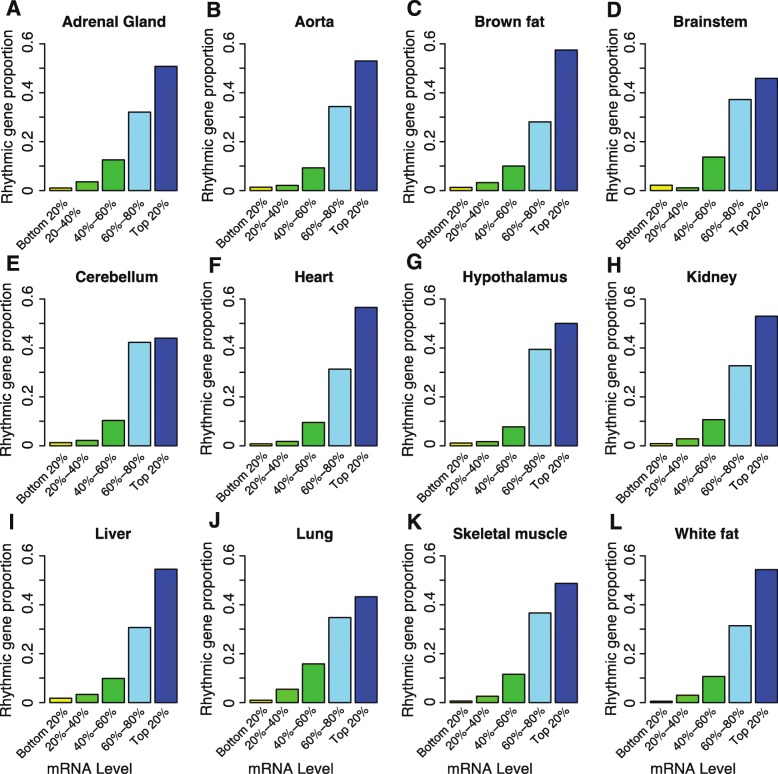
Proportion of rhythmic genes increases with increasing transcription. All genes expressed in each organ were divided into five groups, namely, bottom 20%, 20–40%, 40–60%, 60–80%, and top 20% expressed genes, with the grouping depending on the mRNA expression level. Different colors represent the proportion of rhythmic genes in different groups. **a** Adrenal gland (*n* = 558). **b** Aorta (*n* = 434). **c** Brown fat (*n* = 1411). **d** Brainstem (*n* = 183). **e** Cerebellum (*n* = 232). **f** Heart (*n* = 1008). **g** Hypothalamus (*n* = 180). **h** Kidney (*n* = 2607). **i** Liver (*n* = 3874). **j** Lung (*n* = 2525). **k** Skeletal muscle (*n* = 347). **l** White fat (*n* = 366). The number of rhythmically expressed genes is noted in parentheses. Proportion of rhythmic genes was calculated by the number of rhythmically expressed genes in each group divided by the total number of rhythmically expressed genes in each organ

First, we investigated the distribution of more than 13,000 rhythmically expressed transcripts in 12 mouse organs [14] and found that their distribution was uneven. Although the numbers of rhythmic genes varied greatly from organ to organ (ranging from 180 rhythmic transcripts in the hypothalamus to 3874 transcripts in the liver), the proportion of rhythmic genes increased with increasing expression level (Fig. 1a–l). For instance, the proportion of rhythmic transcripts in the top 20% of most highly expressed genes was 30 times higher than that for the bottom 20% of the least expressed genes in the liver (30% vs. 1%); this ratio was 100 in white fat (2.8% vs. 0.028%). This indicated that highly expressed genes have a strong tendency to be regulated by the circadian network.

**Fig. 2:**
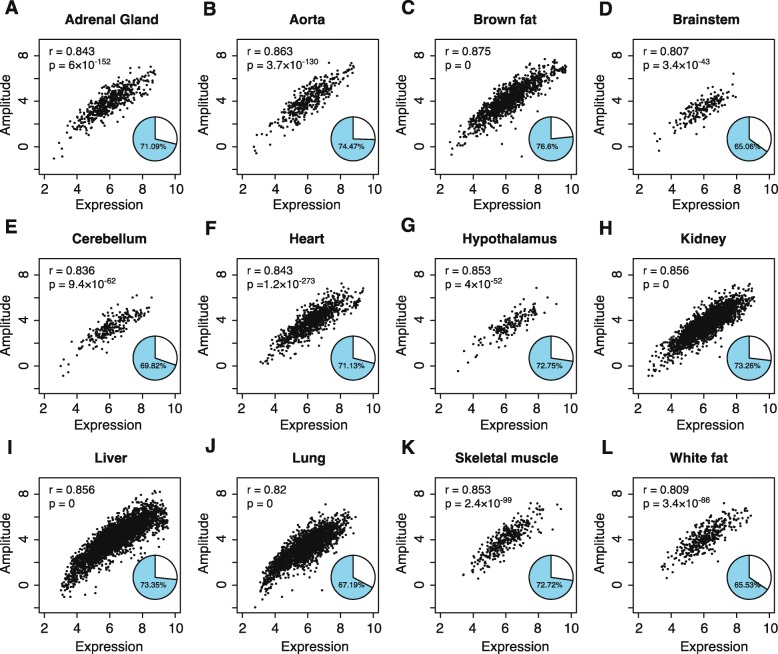
The amplitude of rhythmic genes strongly correlates with their transcriptional level. Scatter plots, characterizes characterizing the relationship between the transcript level and the amplitude of the rhythmic genes, are shown for the different organs. **a** Adrenal gland (*n* = 558). **b** Aorta (*n* = 434). **c** Brown fat (*n* = 1411). **d** Brainstem (*n* = 183). **e** Cerebellum (*n* = 232). **f** Heart (*n* = 1008). **g** Hypothalamus (*n* = 180). **h** Kidney (*n* = 2607). **i** Liver (*n* = 3874). **j** Lung (*n* = 2525). **k** Skeletal muscle (*n* = 347). **l** White fat (*n* = 366). Amplitude is shown log2-transformed; r represents the Pearson correlation coefficient and *p* represents the significance level. Pie chart in each plot shows the percentage of amplitude variation explained by the expression level of rhythmic genes (R^2^)

Since the proportion of rhythmic genes increased with the increasing expression levels, we tested the hypothesis that the transcriptional level of these genes is directly related to their amplitude, defined as the oscillation strength of each cycling gene. Unexpectedly, we observed a strong linear correlation between gene transcriptional level and amplitude (*P* < 1 × 10^− 40^ in all organs; Fig. 2a–l). The strongest correlation was apparent in brown fat and the lowest in the brainstem (*r* = 0.87 and 0.80, respectively; Fig. 2c and d), indicating that 65 to 76% of the variation of amplitude (71% on an average) could be explained by the transcriptional level (Fig. 2a–l). A strong correlation was also apparent when only the top 50% of the highly expressed genes were included in the analysis, indicating that the observation was not an artifact of the noise of low-abundance transcripts (Additional file 1: Figure S1).

Microarray data were used for the computational analyses since the sampling frequency of each tissue was close to that recommended in the large-scale analysis of rhythmic expression [20]. The obtained results were very similar if we use RNA sequencing data from the mouse circadian atlas for the analysis (Additional file 2: Figure S2). In addition to JTK_Cycle, which was originally used to calculate the oscillation features in the mouse atlas, we recalculated the properties of rhythmic genes by using ARSER. The obtained results were similar to those described above (Additional file 3: Figure S3).

### Gene functionality and tissue specificity are not strong predictors of amplitude

**Fig. 3:**
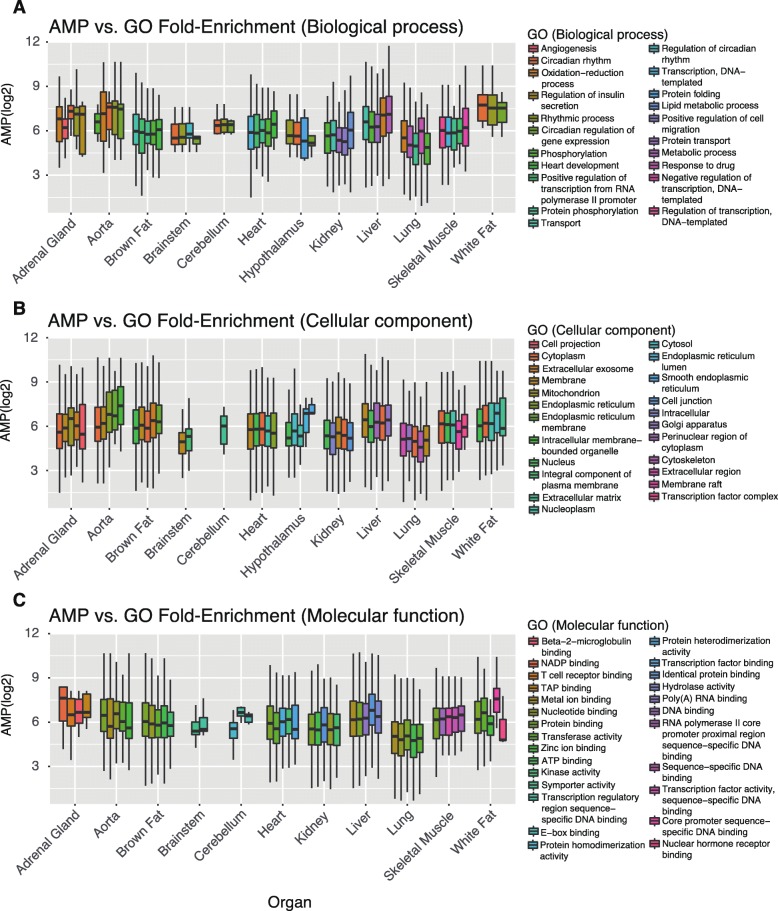
Relationship between functional enrichment and amplitude of rhythmic genes. **a** Biological process. **b** Cellular component. **c** Molecular function. The top 5 significantly enriched (adj. *P* < 0.05) functional annotations were plotted, with the fold-enrichment increasing from left to right in each organ. Different colors represent different functional units, which are annotated on the right in each panel. Amplitude was log2-transformed

It is generally assumed that the function of rhythmically expressed genes is important for their rhythmicity [21, 22]. To test this hypothesis, we explored the relationship between the functional classification of genes, i.e., Gene Ontology (GO), Kyoto Encyclopedia of Genes and Genomes (KEGG), or Reactome annotation terms, and the cycling amplitude of these genes [23, 24]. Generally, the amplitude of rhythmic genes did not obviously increase with the increase of the fold-enrichment of functional pathways and did not consistently increase with the enrichment significance (Additional file 4: Table S1 and Table S2). Further, in 10 out of 12 organs, the average amplitude of rhythmic genes in the most enriched pathways was not the highest for the annotation from biological process among all the pathways examined (10/12 for the annotation of cellular components and 6/12 for the annotation of molecular function) (Fig. 3). In addition, no significant correlation existed between the pathway fold-enrichment and amplitude among the top 5 most-enriched pathways classified by the GO annotation as “cellular component” or the remaining four annotations (“biological process”, “molecular function”, KEGG, or Reactome pathways, *P* > 0.05) (Fig. 3 and Additional file 4: Table S3). We found that this relationship extended to the enriched pathways in all (12) organs but was slightly significantly anti-correlated in Reactome pathway analysis (*r* = 0.005 for biological process; *r* = 0.04 for cellular component; *r* = − 0.029 for molecular function; and *r* = − 0.25 for Reactome pathway analysis; Additional file 4: Table S4). Finally, the correlation between the mean amplitude and fold-enrichment of all the cycling gene related pathways is not high, after controlling for expression level (R^2^ < 0.1 in all the tissues, partial correlation test). (Additional file 4: Table S5). Collectively, these data indicated that although the functionality and rhythmicity of the genes are linked, the functionality is not a strong predictor of amplitude compared with the gene transcriptional level.

Further, we found that tissue specificity was correlated with amplitude, i.e., the amplitude of genes that were rhythmically expressed in multiple organs was greater than that of genes rhythmically expressed in only one organ. The correlation between the two parameters was moderate in the 12 organs analyzed (*r* = 0.26 on average, *P* < 0.05 in all organs; Additional file 5: Figure S4*A*–*L*). Further, the expression levels of genes that were cyclically expressed in multiple organs were higher in some organs than in others (Additional file 6: Figure S5*A*–*L*). By utilizing partial correlation analysis, we also observed that the effect of tissue specificity did not explain the correlation between the transcriptional level and amplitude (0.81 ≤ *r* ≤ 0.88 after controlling for the effect of cyclic tissue number; Additional file 4: Table S6). These observations indicated that tissue specificity is a positive but not strong predictor of amplitude compared with the transcriptional level. In addition, we have compared the housekeeping genes with other genes and little differences between the amplitude of cycling housekeeping genes and other cycling genes were found, suggesting that housekeeping gene is not a strong predictor of cycling genes (Additional file 4: Table S7).

### Energetic cost is linked to expression level and explains the strength of circadian oscillation

Since the transcription of each gene is not cost free, highly expressed genes require greater energy expenditure during transcription than genes expressed at a lower level. Downregulation of the expression of these genes when they are not needed serves to reduce the overall metabolic cost in the cell [25]. We next determined the synthetic cost of the rhythmic transcripts. Briefly, the energetic cost of each mRNA molecule was calculated based on the sequence composition by integrating the energy required for the precursors during mRNA synthesis, the energy required for transcription initiation and termination, and the rate of mRNA degradation. The total energy cost of the transcription of each gene was calculated taking into account the mRNA decay rate and the transcriptional level [25, 26]. As anticipated, we observed a strong positive correlation between the expression level of the transcripts and energy consumption during their transcription (*r* > 0.75, *P* < 1 × 10^− 50^ in all organs; Additional file 7: Figure S6), implying that the rhythmical regulation of transcription of highly expressed genes also determines the energy expenditure.

**Fig. 4:**
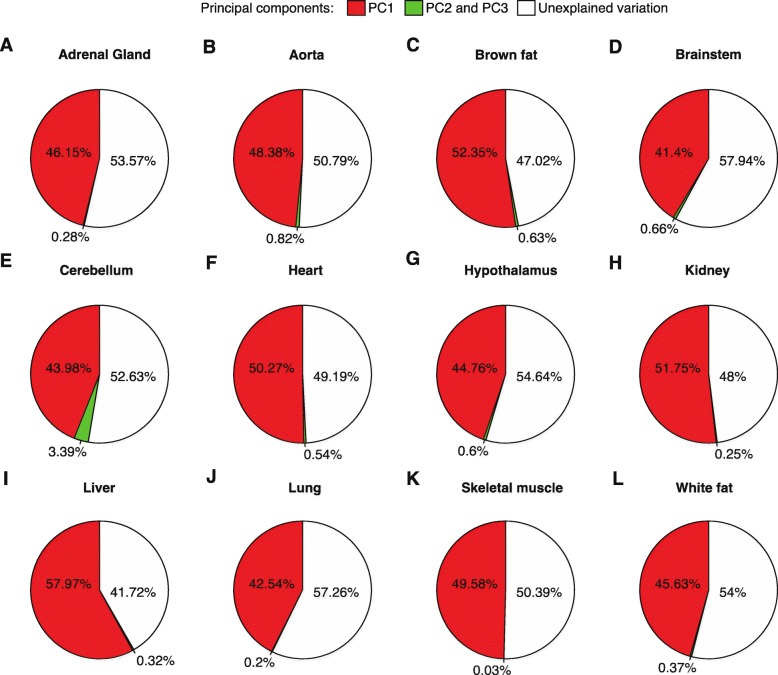
Variation of amplitude explained by the energetic cost. As shown, 40 to 60% of the variation of amplitude can be explained by the energetic cost of rhythmic genes. PC1 contributes the most to the amplitude variation, while PC2 and PC3 contribute very little. **a** Adrenal gland (*n* = 558). **b** Aorta (*n* = 434). **c** Brown fat (*n* = 1411). **e** Brainstem (*n* = 183). **e** Cerebellum (*n* = 232). **f** Heart (*n* = 1008). **g** Hypothalamus (*n* = 180). **h** Kidney (*n* = 2607). **i** Liver (*n* = 3874). **j** Lung (*n* = 2525). **k** Skeletal muscle (*n* = 347). **l** White fat (*n* = 366). Pie charts represent the percentage of amplitude that can be explained by each principal component. Red, percentage of variation explained by PC1; green, percentage of variation explained by the PC2 and PC3; white, unexplained variation

In addition, we found that although the amounts of energy consumed during the transcription of 5′ UTR, 3′ UTR, and coding regions are different by several orders of magnitude, they correlated with the amplitude of rhythmic transcripts (*r* > 0.4, *P* < 1 × 10^− 8^ in all comparisons; Additional file 4: Table S8). This correlation was stronger for the 5′ UTR region than for the 3′ UTR region (Additional file 4: Table S8). According to the linear model, the energy cost of 5′ UTR could explain 31 to 44% of the variation of amplitude; that of 3′ UTR, 19 to 39% variation; and that of coding region, 31 to 49% variation (Additional file 4: Table S8). Since the three factors are inter-correlated (*r* > 0.7, *P* < 1 × 10^− 100^ in all comparisons; Additional file 4: Table S9), we then performed principal component analysis to evaluate the overall effect of these factors on amplitude. We found that the first principal component (PC1) accounted for the most variation, explaining 41.4 to 58.0% (48% on average) of the variation of amplitude, while PC2 and PC3 explained only very low percentages of variation in each organ (Fig. 4 and Additional file 8: Figure S7). Finally, we permuted the amplitude and repeated the analysis model of principal components 1000 times. We found that the explained variations of amplitude were significantly higher than those identified in permutation experiments (*P* < 0.001). Collectively, the presented results indicate the importance of the regulation of energetic cost for rhythmic gene transcription.

## Discussion

In mammals, over 50% of the transcriptome is rhythmically regulated in at least one organ. Although previous studies have shown that the expression level of rhythmically expressed genes is higher than that of other genes [25, 27, 28], the extent to which this factor contributed to the rhythmicity of gene expression remained unclear. This question is important considering the possibility of existence of other factors such as different functional pathways that might govern the expression rhythmicity, should the expression level exert only a minor effect on rhythmic gene expression. In the current study, we showed that the expression level of transcripts plays a crucial role in determining whether the rhythmic transcripts are regulated by the circadian regulatory network or not, and that the effect of expression level exceeds that of other potential factors, such as functionality and tissue specificity. We further showed that this single factor can explain > 70% of the variation in the amplitude of rhythmic transcripts. Further, the higher the expression of rhythmic genes, the greater the energy expenditure of the transcription process. Transcriptional systems tend to downregulate the highly expressed genes when their function is not necessary.

Circadian rhythms are closely linked with the cellular metabolism [2, 29]. For instance, the activity of BMAL, one of the core regulators of the circadian regulatory network, is regulated by the transcriptional repressor REV-ERB [30, 31]. The findings of the current study suggest that the output of the circadian regulatory network itself is an energy-saving strategy for the gene expression process. Collectively, these lines of evidence indicate that the regulation of metabolism and metabolic cost are critical for the evolutionary adaptation of the cell.

The lack of preservation of rhythmic properties among diverge tissues and organs, or between divergent species, is different from that of gene function, as the latter is typically highly evolutionarily conserved. This difference is a strong indication that the direct link between the rhythmicity and functionality of the cyclic genes is very weak. Since the regulation of highly expressed genes is a major requirement for circadian gene expression, functional pathways that contain many highly expressed genes are usually over-enriched in cyclic genes, compared with pathways that are expressed relatively weakly. Pathway analysis of rhythmic gene expression should take into account the effects of gene expression levels to obtain an unbiased view of the functional distribution of those genes, as a warning for interpreting many previous cyclic pathway analyses.

The observations of the current study indicate that a specific biological function plays a minor role in determining the rhythmic gene expression. Since selective downregulation of highly expressed genes is a systematic strategy to reduce the energetic cost of transcription, undoubtedly, under some specific circumstances, the function of a particular gene could be directly related to its rhythmicity. For example, *PER1*, *PER2*, and *PER3* are expressed periodically in at least 8 of 13 human tissues [16], and *Per2* shows robust rhythmic transcription in the mouse liver [12].

The findings of the current study also indicate that identification of genes whose function is directly related to their rhythmic expression pattern is not a trivial task. Two potential approaches are proposed here for further consideration. One involves controlling for the effect of gene expression, as the expression level is the primary factor determining the rhythmic expression of cyclic genes. If the expression profile of a particular gene is robustly rhythmic regardless of whether the gene is overexpressed or underexpressed in a cell, the function of the gene may be related to its rhythmicity. Another approach is controlling for tissue specificity. We are convinced that genes that are rhythmically expressed in multiple tissues are most likely to be strong candidates for essential cyclic genes. Ultimately, one may find that, contrary to the current widespread observations that the majority of transcribed genes are rhythmically expressed, only a small fraction of these genes are essential cyclic genes.

## Conclusions

We here showed that the transcriptional level is the single factor that dominates the behavior of rhythmic genes in mouse organs. In mouse, on the molecular level, the circadian regulatory network mainly regulates highly expressed genes rather than other genes, to reduce the overall energetic cost. Although many key genes influencing the circadian behavior have been identified in the past decades, big gaps still exist to obtain a full explanation of the circadian behavioral phenotypes based on the underlying plethora of molecular activities.

## Methods

### Data collection

The expression profiles of all transcripts from 12 organs of mouse (*Mus musculus*) were derived from the mouse circadian gene expression atlas (last accessed on August, 2018), which is currently the largest repository for rhythmic expression data for mouse [14]. The parameters of rhythmic gene expression were calculated by using JTK_ Cycle [32, 33], with adjusted *P* < 0.05 (Benjamini-Hochberg–corrected) as the cutoff for identifying cycling genes. Amplitude refers to a one-cycle median sign-adjusted deviation from the median expression, and was calculated by using JTK_Cycle. Only genes with assigned expression values across all time points in a particular tissue were considered “expressed” and used in the analysis. Almost half of the expressed transcripts are rhythmically transcribed [14]. Finally, similar parameters were calculated by ARSER to double check the primarily results. The parameters for determining the energetic cost of each mRNA molecule, such as the synthesis energy required, were derived from the determinations for the yeast metabolic system and based on the number of activated phosphate bonds (~P) [26]. The genome-wide mRNA degradation rates were determined by metabolic pulse labeling, as previously reported [34]. The analyses were made under the assumption that the degradation rate is primarily determined by the mRNA sequence and relatively consistent at different rhythmic time points.

### Functional analysis

To determine whether functional classification exerts a dominant influence on the amplitude of rhythmic genes, enrichment analysis of cycling genes in each organ was performed by utilizing clusterprofiler [35] and the database for annotation, visualization, and integrated discovery (DAVID) (https://david.ncifcrf.gov/) [36, 37] and the Reactome pathway website (https://reactome.org/) [38, 39]. GO analysis (enrichment for “Biological process”, “Cellular component”, and “Molecular function”) and KEGG pathway analysis were performed by using the former; Reactome biological pathways were analyzed by using the latter. Background gene list was set containing all the expressed genes in each tissue. Both fold-enrichment value and significance value (*p*) from the analysis were used as indicators for the strength of cycling gene enrichment in a specific functional category. Log (*p*) values were used to facilitate downstream analysis. Finally, for each enriched pathway, the average amplitude of rhythmic genes was calculated and correlated with the enrichment strength by using R codes. For instance, for the “Biological process” analysis in liver, all the 5822 terms were considered as the background functional term list, and all the 2632 cycling genes detected in liver were used to search for the enriched pathways, with multiple testing correction. The results show that 499 pathways were enriched (*P* < 0.05, BH-correction). Other enrichment analyses were performed similarly.

### Calculation of the energetic cost of mRNA

The energetic cost of mRNA was determined by the amount of activated phosphate bonds (~P) as described previously [25, 26]. The synthesis cost for each mRNA molecule is mainly determined by the energy usage of synthesizing each nucleotide and the nucleotide composition of mRNA. Hence, both the synthesis cost of single mRNA molecule and its copy number were considered in each calculation. To distinguish the cost effects of different transcriptional regions on the amplitude, the energetic costs of 3′ UTR, 5′ UTR, and coding regions were calculated separately. Overall, 30,720,384 transcripts were analyzed. The cost for each mouse gene is listed in Additional file 9: Table S10.

### Linear regression analysis

Linear regression analysis was used to quantifying the relationship between the transcription level and amplitude of rhythmic genes. Averaged expression from different sampling time points was calculated for each organ. Logarithm of the averaged expression was then correlated with the logarithm of amplitude value of each rhythmic gene. To describe the extent to which the changes in expression affected the changes in amplitude, the coefficient of determination in the linear regression was calculated. As in a typical common interpretation of linear regression analysis, R^2^ was used to indicate the contribution of the transcription level to the variance of amplitude, namely*,* the explained variation of the amplitude of rhythmic genes.

To examine whether the proportion of cycling genes increases with the increasing transcription, the average expression levels in each organ were used. All the expressed genes in each organ were divided into five groups according to expression level (top 20%, 20–40%, 40–60%, 60–80%, and bottom 20%). The proportion of cycling genes in each category was calculated as the number of cycling genes in that category divided by the total number of cycling genes. Following this strategy, “top 50% highly expressed genes” was defined as the top half of all genes with assigned expression values at all sampling time points in each tissue. This gene set was then used to determine the existence of correlation between the expression level and amplitude of highly expressed genes.

### Principal component analysis

Principal component analysis was used to evaluate the overall contribution of energetic cost to the amplitude of rhythmic genes. That was because although the energetic cost of 5′ UTR, 3′ UTR, and the coding region of rhythmic transcription strongly correlated with amplitude, these three variables were also significantly interrelated. The dimensionality of these factors was reduced by using the principal component analysis [40]. The analysis was performed using the formula: amplitude ~ cost of 5′ UTR + cost of 3′ UTR + cost of coding region. Overall, all genes containing 5′ UTR and 3′ UTR regions (19,622 genes) were included in the analysis. In the permutation experiments, the amplitude value of rhythmic genes was shuffled 1000 times; for each time point, the principal component analysis was performed, and the explained effect of energetic cost on amplitude was determined. Energetic cost and amplitude values were log-transformed for all the above analyses.

### Partial correlation test

Analyses indicated that for rhythmic genes, the amplitude slightly increases as the cyclic tissue number increases. Cyclic tissue number was defined as the number of tissues in which a particular transcript exhibits rhythmic expression. To investigate whether the correlation between transcription level and amplitude existed after controlling for this effect, a partial correlation test was used. Partial correlation coefficients were calculated for each organ [41].

### Statistical analysis

The analysis and processing of all the data were performed by using R software. The “stats” package was used for the linear regression analysis and principal component analysis, and “ggm” package was used for the partial correlation test.

## Supplementary information

**Additional file 1: Figure S1.** The amplitude of rhythmic genes in top 50% expressed genes strongly correlates with their transcriptional level.

**Additional file 2: Figure S2.** The RNA sequencing data results were very similar to those for the analysis of microarray data from the mouse circadian atlas.

**Additional file 3: Figure S3.** The ARSER results were similar to those from JTK_Cycle.

**Additional file 4: Table S1.** Relationship between rhythmic transcript enrichment pathways (fold-enrichment) and their mean amplitude. **Table S2.** Relationship between rhythmic transcript enrichment pathways (significance level, *P*-value) and their mean amplitude. **Table S3.** Relationship between the top 5 rhythmic transcript enrichment pathways (fold-enrichment) and their mean amplitude in all organs. **Table S4.** Relationship between all the rhythmic transcript enrichment pathways (fold-enrichment) and their mean amplitude. **Table S5.** Partial correlation between the mean amplitude and fold-enrichment of the cycling gene related pathways, after controlling for expression level. **Table S6.** Partial correlation between transcription level and amplitude of rhythmic genes, after controlling for cyclic tissue number. **Table S7.** Difference between amplitude of cycling housekeeping genes and other cycling genes. **Table S8.** Correlation between the energetic cost and amplitude for the 5′ UTR, 3′ UTR, and coding region of rhythmic transcripts. **Table S9.** The energetic costs of 5′ UTR, 3′ UTR, and coding region of rhythmic transcripts are highly inter-correlated.

**Additional file 5: Figure S4.** Relationship between the amplitude of rhythmic genes and the number of tissues in which they are rhythmically expressed.

**Additional file 6: Figure S5.** Relationship between transcriptional level of rhythmic genes and the number of rhythmically expressed tissues.

**Additional file 7: Figure S6.** Highly expressed rhythmic genes are more expensive than other genes.

**Additional file 8: Figure S7.** PC1 from the principle component analysis of energetic cost (5′ UTR, 3′ UTR, and coding regions) is strongly correlated with amplitude.

**Additional file 9: Table S10.** The cost for each mouse gene

## Data Availability

The expression profiles of all transcripts from 12 mouse organs were from GEO database and the accession numbers is GSE54652. All codes are available from the corresponding authors upon request.

## Abbreviations

Adr: Adrenal gland
BFAT: Brown fat
BP: Biological process
BS: Brainstem
CC: Cellular component
Cere: Cerebellum
Hypo: Hypothalamus
MF: Molecular function
Mus: Skeletal muscle
WFAT: White fat
