# Supplementary figures and images for: A single factor dominates the behavior of rhythmic genes in mouse organs
Source: BMC Genomics. 2019 Nov 20;20:879. doi: 10.1186/s12864-019-6255-3 (PMC6868821; doi:10.1186/s12864-019-6255-3)

## Slide 1
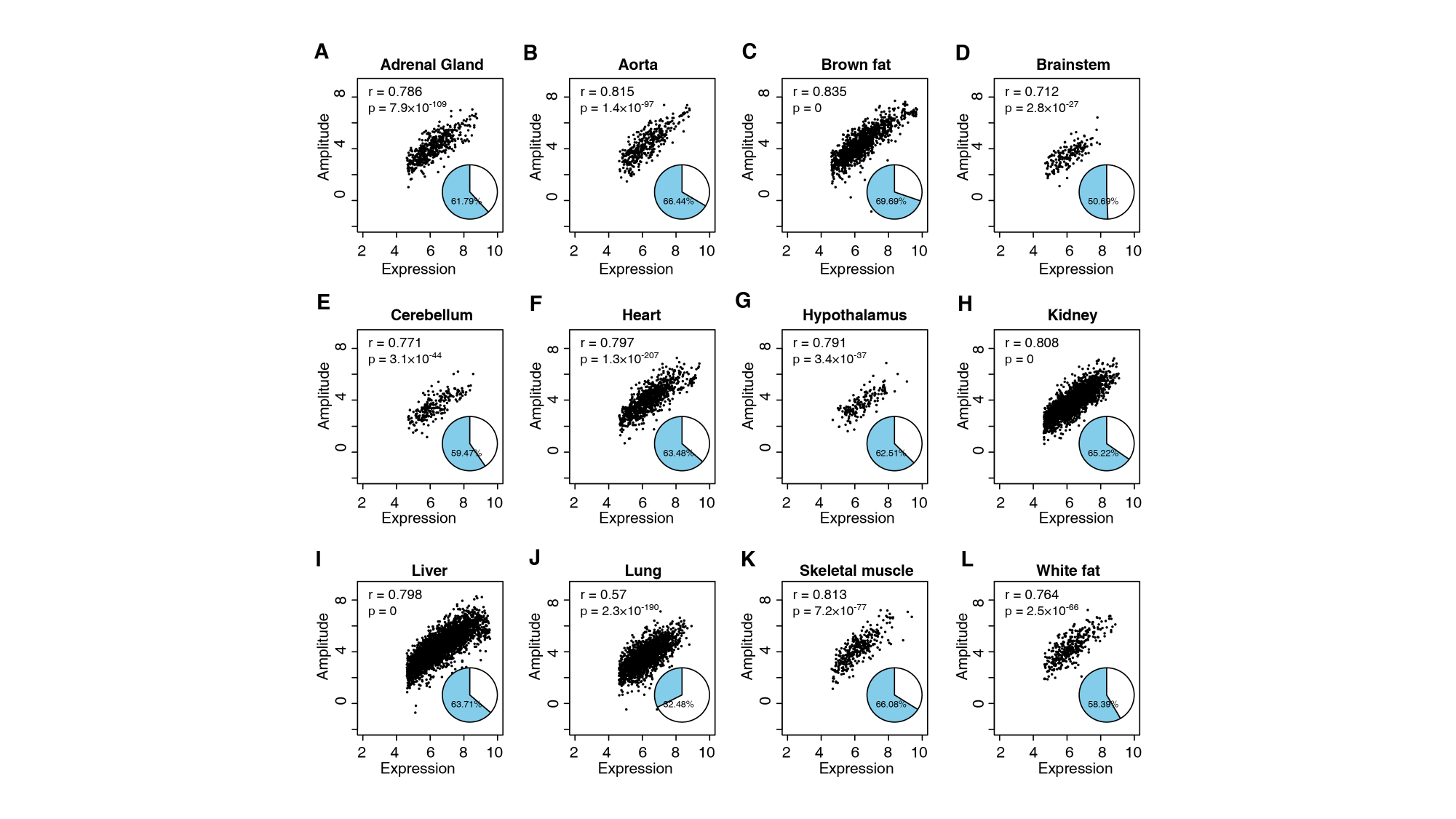

Supplement: Supplementary file 1 — Additional file 1: Figure S1. The amplitude of rhythmic genes in top 50% expressed genes strongly correlates with their transcriptional level. [file 12864_2019_6255_MOESM1_ESM.pptx]

## Slide 1
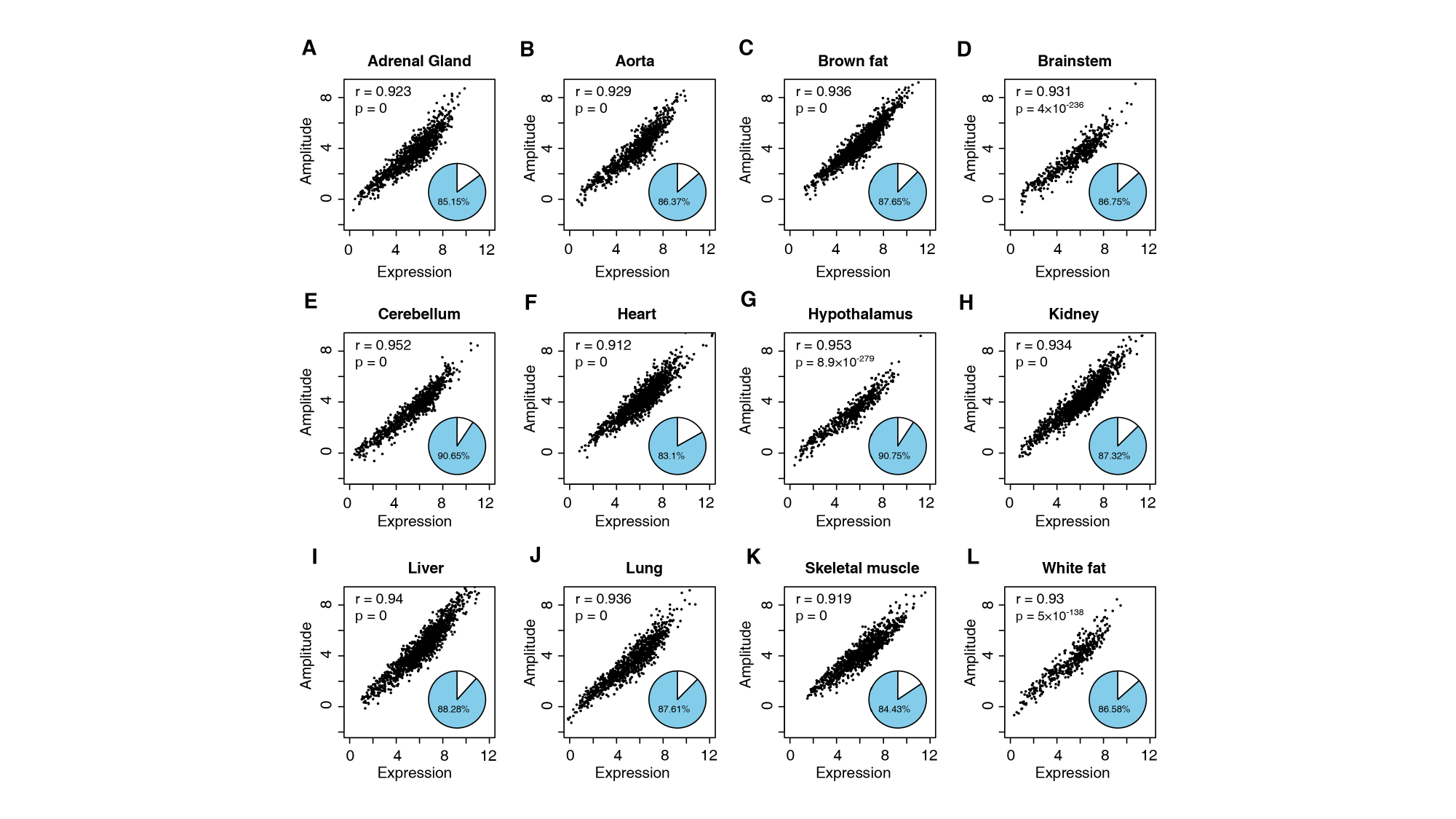

Supplement: Supplementary file 2 — Additional file 2: Figure S2. The RNA sequencing data results were very similar to those for the analysis of microarray data from the mouse circadian atlas. [file 12864_2019_6255_MOESM2_ESM.pptx]

## Slide 1
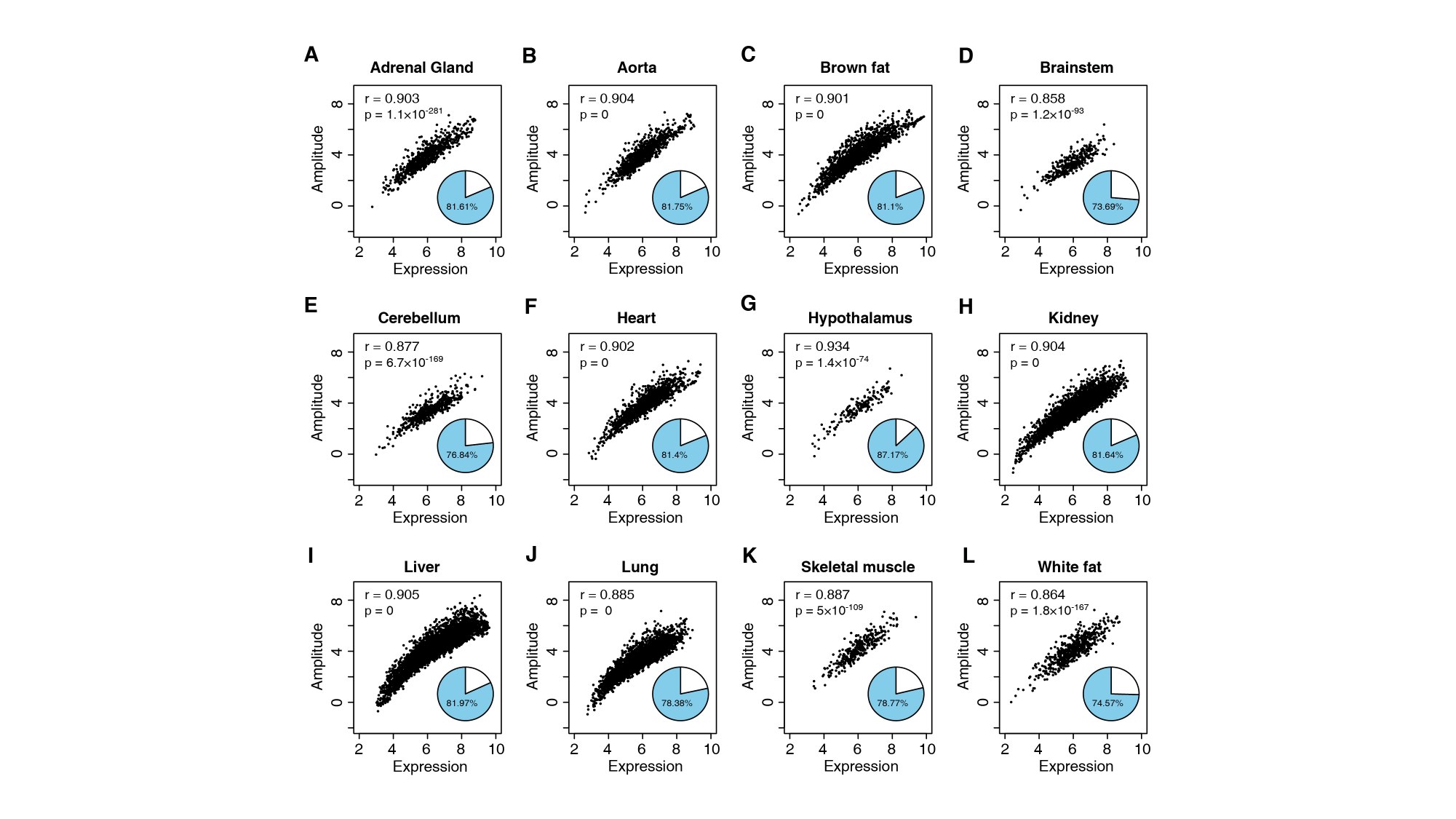

Supplement: Supplementary file 3 — Additional file 3: Figure S3. The ARSER results were similar to those from JTK_Cycle. [file 12864_2019_6255_MOESM3_ESM.pptx]

## Slide 1
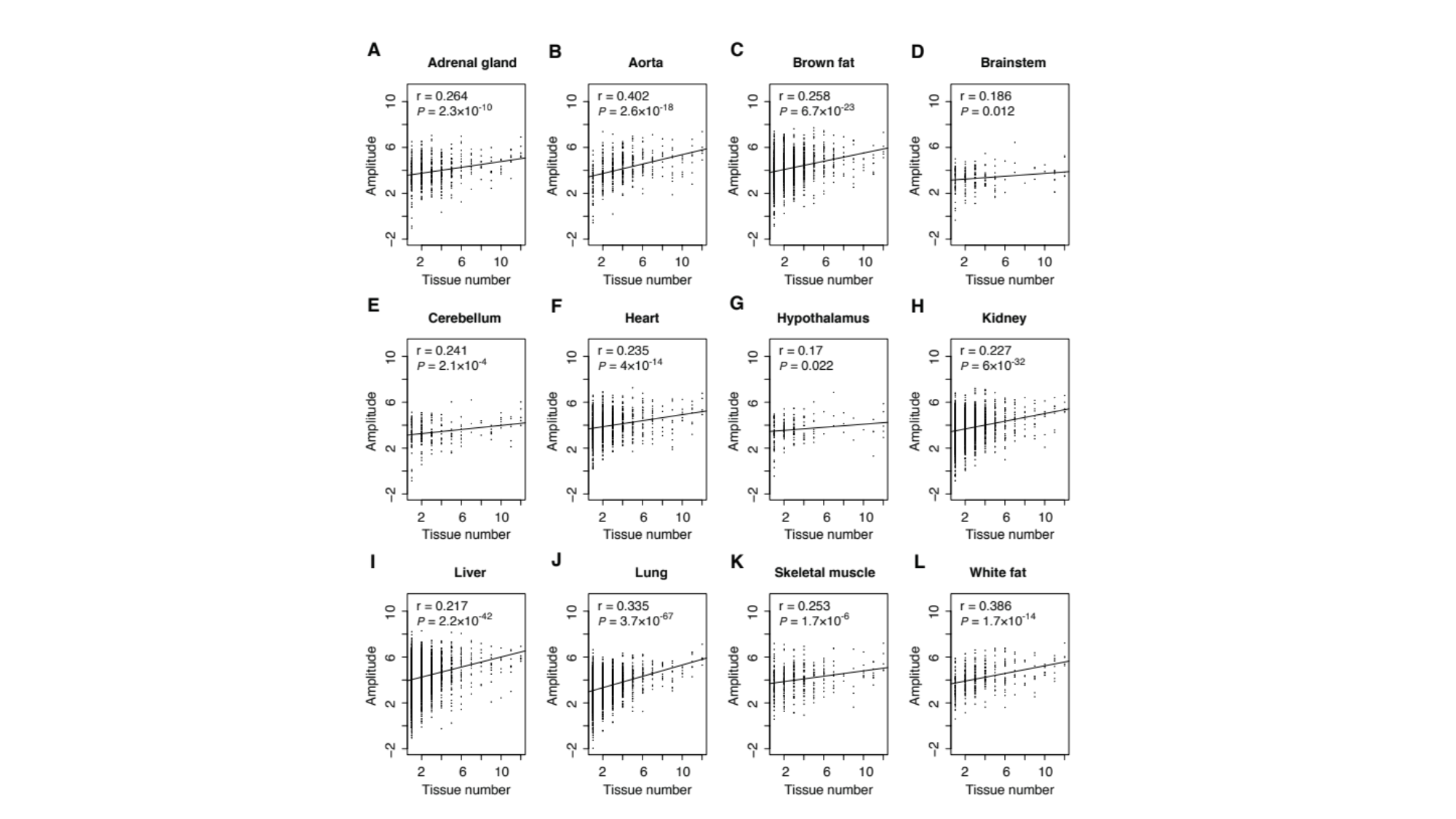

Supplement: Supplementary file 4 — Additional file 4: Table S1. Relationship between rhythmic transcript enrichment pathways (fold-enrichment) and their mean amplitude. Table S2. Relationship between rhythmic transcript enrichment pathways (significance level, P-value) and their mean amplitude. Table S3. Relationship between the top 5 rhythmic transcript enrichment pathways (fold-enrichment) and their mean amplitude in all organs. Table S4. Relationship between all the rhythmic transcript enrichment pathways (fold-enrichment) and their mean amplitude. Table S5. Partial correlation between the mean amplitude and fold-enrichment of the cycling gene related pathways, after controlling for expression level. Table S6. Partial correlation between transcription level and amplitude of rhythmic genes, after controlling for cyclic tissue number. Table S7. Difference between amplitude of cycling housekeeping genes and other cycling genes. Table S8. Correlation between the energetic cost and amplitude for the 5′ UTR, 3′ UTR, and coding region of rhythmic transcripts. Table S9. The energetic costs of 5′ UTR, 3′ UTR, and coding region of rhythmic transcripts are highly inter-correlated. [file 12864_2019_6255_MOESM4_ESM.pptx]

## Slide 1
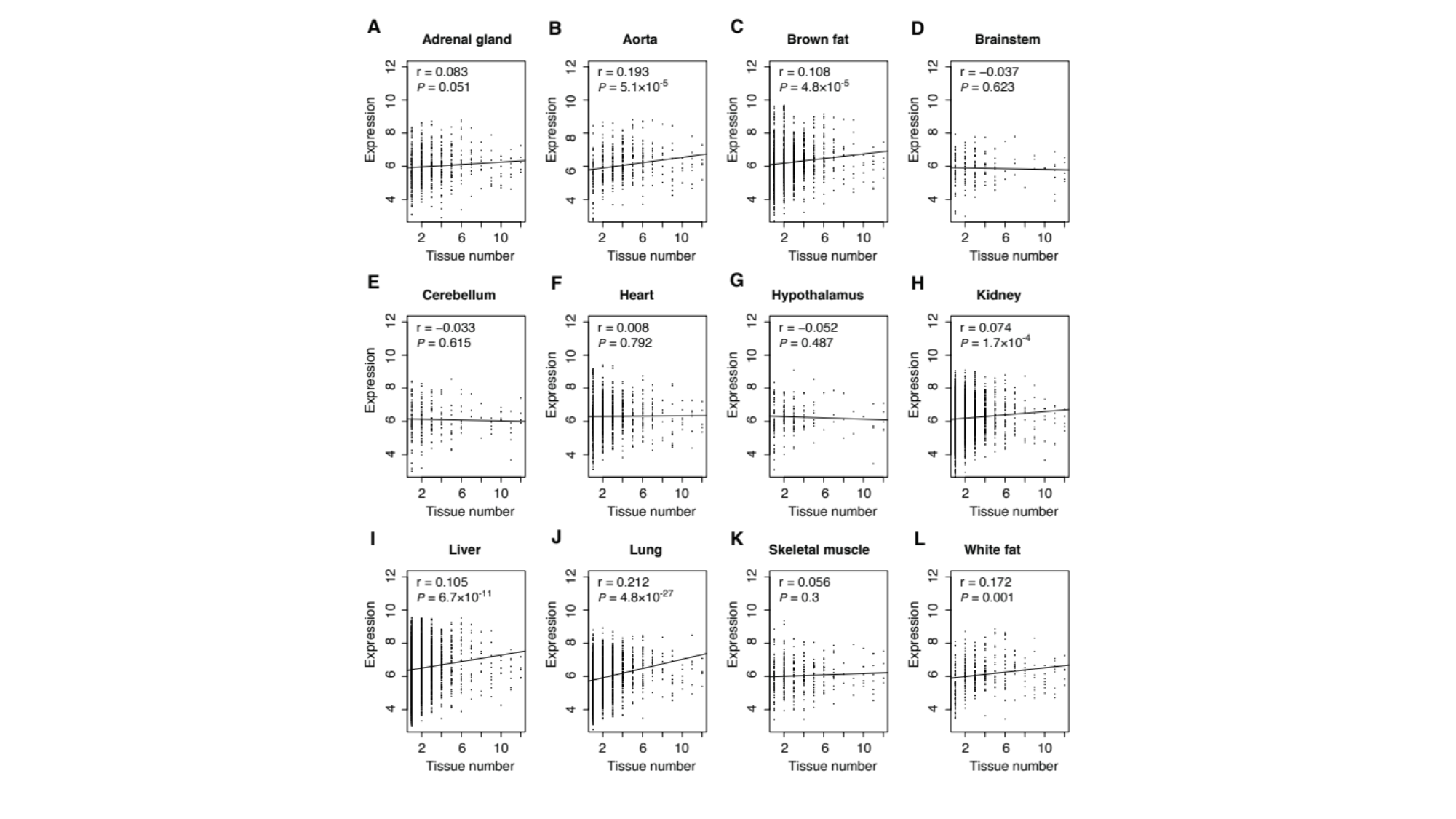

Supplement: Supplementary file 5 — Additional file 5: Figure S4. Relationship between the amplitude of rhythmic genes and the number of tissues in which they are rhythmically expressed. [file 12864_2019_6255_MOESM5_ESM.pptx]

## Slide 1
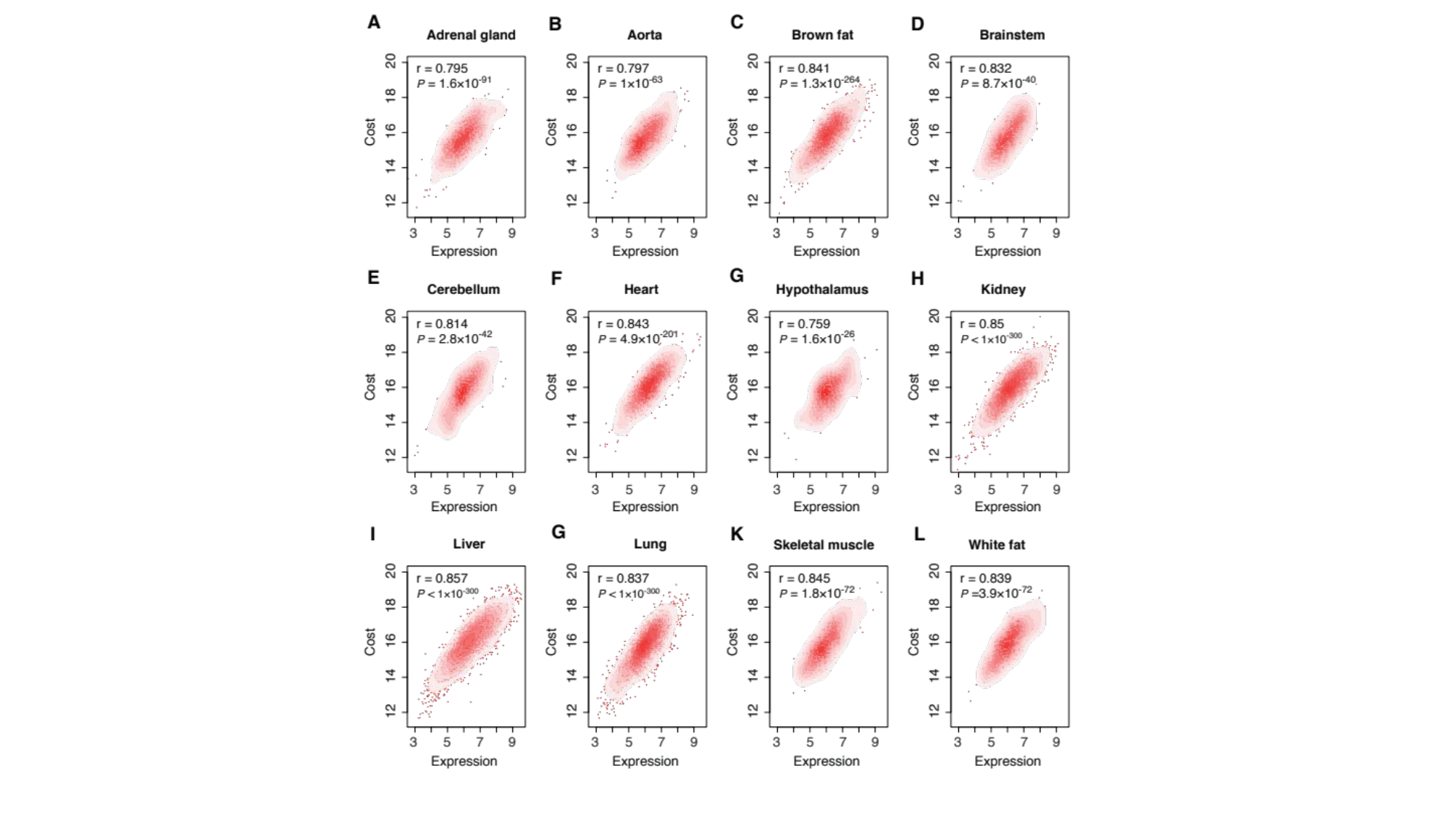

Supplement: Supplementary file 6 — Additional file 6: Figure S5. Relationship between transcriptional level of rhythmic genes and the number of rhythmically expressed tissues. [file 12864_2019_6255_MOESM6_ESM.pptx]

## Slide 1
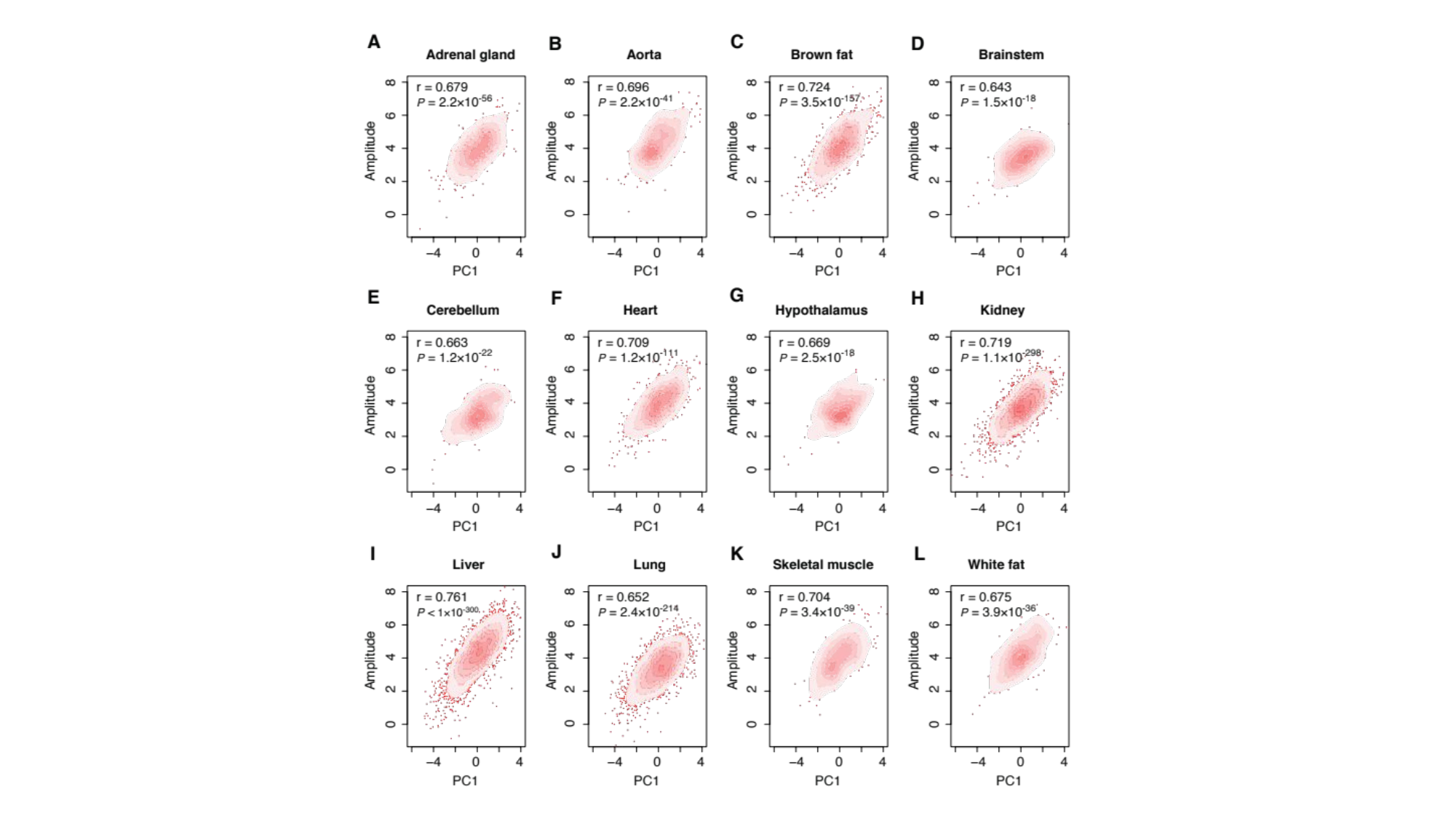

Supplement: Supplementary file 7 — Additional file 7: Figure S6. Highly expressed rhythmic genes are more expensive than other genes. [file 12864_2019_6255_MOESM7_ESM.pptx]
